# Neuroticism as Mediator and Moderator Between War Atrocities and Psychopathology in Syrian Refugee Children and Adolescents

**DOI:** 10.3389/fpsyg.2022.811920

**Published:** 2022-01-27

**Authors:** Vivian Khamis

**Affiliations:** Department of Education, Faculty of Arts and Sciences, American University of Beirut, Beirut, Lebanon

**Keywords:** war atrocities, neuroticism, posttraumatic stress disorder, emotion dysregulation, Syrian refugee children and adolescents, behavioral and emotional disorders, Lebanon, Jordan

## Abstract

**Background:**

Despite the extensive research on war atrocities and risk factors for psychopathology, there is a paucity of research on the potential mediating and moderating effect of neuroticism in refugee children and adolescents.

**Objective:**

This study aimed to analyze whether neuroticism mediated and/or moderated the relationship between war atrocities and different types of psychopathology in Syrian refugee children and adolescents who resettled in Lebanon and Jordan.

**Participants and Setting:**

Participants were 1,000 Syrian refugee children and adolescents of both sexes.

**Methods:**

Questionnaires were administered in an interview format with children at school by two trained psychologists. Descriptive statistics and inter-correlations among variables were used Then the mediator and moderator effect of neuroticism in the relationship between war atrocities and posttraumatic stress, emotion regulation and behavioral and emotional disorders were examined.

**Results:**

A partial mediating effect of neuroticism on posttraumatic stress disorder (PTSD), emotion dysregulation, and emotional and behavioral disorders was revealed. Findings also indicated full moderating effects of neuroticism on PTSD as well as partial moderating effects on emotion dysregulation, and emotional and behavioral.

**Conclusions:**

Findings contribute to the neuroticism literature by showing that high-N children develop more problems that are psychopathological and have more severe affective reactions to war atrocities in post- resettlement contexts. Early interventions aimed to reduce neuroticism might contribute to a better prognosis in refugee children at high-risk for psychological disorders.

## Introduction

The escalation of armed conflict in Syria brought with it a great upsurge of interest, particularly among psychologists and psychiatrists, in studying the impact of war trauma on refugees. Exposure to terrorism, war and political violence are highly pervasive experiences that have deleterious ramifications on children and adolescents (e.g., Khamis, 2005, 2008, 2012; Masten and Narayan, 2012). Much evidence exists linking war trauma to negative psychological outcomes, such as posttraumatic stress disorder, depression, and anxiety (Fazel et al., 2012; Khamis, 2019). Conditional risk of undesirable developmental outcomes in children is highly variable by both dispositional and situational variables that play a role in the traumatic stress and coping process. The personality variable that has received the most attention with respect to stress and coping is neuroticism (e.g., Bolger and Zuckerman, 1995). Neuroticism represents a continuum of individual differences, with low levels representing emotional stability and even temperedness, and high levels representing negative emotionality (John et al., 2008). It has been proposed that neuroticism may act as a vulnerability factor in the development of all forms of psychopathology (Santor and Rosenbluth, 2005; Kotov et al., 2010). For example, high levels of neuroticism have been related to undesirable mental health outcomes such as posttraumatic disorder (Hasanović, 2012; Breslau and Schultz, 2013), and emotion dysregulation (Kokkonen and Pulkkinen, 2001; Weinstock and Whisman, 2006; Mącik et al., 2019). Further, high neuroticism characterize children at risk for emotional and behavioral disorders (e.g., Khan et al., 2005; Lahey, 2009), including oppositional defiant disorder (ODD) and nattention and/or hyperactivity-impulsivity (ADHD) (Martel and Nigg, 2006).

The present research was designed to broaden our understanding of the impact of war atrocities on different mental health outcomes in refugee children by prospectively examining the potential mediator and/or moderator effect of neuroticism. If neuroticism symptoms mediate the relationship between war atrocities and childhood posttraumatic stress, emotion dysregulation and behavioral and emotional problems, then the later relationship should become non-significant when neuroticism symptoms are controlled. If neuroticism symptoms moderate childhood posttraumatic stress, emotion dysregulation and behavioral and emotional problems, then such a relationship should be attenuated for children with low levels of neuroticism symptoms and strengthened for children with high levels of neuroticism symptoms. The elucidation of mediating and moderating factors provides the building blocks in the identification of pathways to resilience in children. Recognition of such variables is important for the development of interventions that focus on the identification and mobilization of adaptive systems within the individual.

## Materials and Methods

### Sample Selection and Procedure

A multistage stratified cluster sample was used to identify 1,000 Syrian refugee children who fled their country during the war and now resided in Lebanon and Jordan. First, children were selected from the two countries (Lebanon and Jordan), representing various governorates, schools, grade level (1–12), and gender (see Khamis, 2019). Of the 1,000 children and adolescents who were selected for entry into the study 130 participants declined to participate and as a result 130 other children and adolescents were selected for replacement. Two trained psychologists administered questionnaires in an interview format with children and adolescents at school. Completion of the interview took from 35 to 45 min. Ethics approval for the study was obtained from the University.

### Participants

A total of 1,000 Syrian refugee school-age children and adolescents participated in this study. The sample consisted of 500 children and adolescents from each of the two countries (Lebanon and Jordan). Children and adolescents from Lebanon were accessed through the public school system and a non-formal education program (Jussor) whereas children and adolescents from Jordan were accessed solely through public schools representing various districts and grade levels (i.e., 1–12). They ranged in age from 7 to 18 years (*M* = 11.30; *SD* = 2.65). Of these children and adolescents, 461 (46.1%) were males and 539 (53.9%) were females. The time these children and adolescents spent in the host country ranged from 1 to 72 months (*M* = 48.64, *SD* = 15.58).

### Measures

#### Demographic Characteristics

Children and adolescents completed a brief questionnaire designed to obtain basic demographic information, including child’s age in years, gender, host country, and time spent in host country since they fled from Syria.

#### War Atrocities

Trauma exposure scale was used to assess the number of war atrocities experienced by children and adolescents during the war (Khamis, 2015). The scale is composed of 17 items scored as 1 “yes” and 0 “no.” Responses are summed to arrive at a total scale score. Scores vary from 0 (no trauma exposure at all) to 18 (high trauma exposure). Examples of the items are a family member, a relative or a close friend was killed during the war, our house was bombarded or destructed, hearing the sounds of rocket attacks, shelling and bombardment, witnessing people injured. Two items were changed to reflect the refugees experience such as heard the sounds of explosions and was forced to live in a refugee camp. Cronbach’s for the total scale in this sample is 0.85.

#### Neuroticism

The neuroticism scale of Eysenck Personality Questionnaire (Eysenck and Eysenck, 1968) assessed neuroticism. Children were asked to complete the 19-item Arabic version of the neuroticism scale (El Khalek, 1978), which asked children to answer yes or no questions about their negative affectivity. Composite scores could range from 0 to 19, with higher scores indicating higher neuroticism. Cronbach’s alpha for the total scale in this sample was 0.87.

#### Posttraumatic Stress Disorder

Posttraumatic stress disorder (PTSD) was assessed by using the diagnostic criteria for an assessment of PTSD as outlined in the DSM-IV (American Psychiatric Association [APA], 1994). A structured clinical interview was used to ensure coverage of all the relevant signs and symptoms of PTSD. The posttraumatic stress disorder module of the structured clinical interview for the DSM-IV has been previously used on children and adolescents in the Arab world, and the inter-rater kappa coefficients measuring the reliability of interviewers was 0.90 for current and lifetime PTSD (Khamis, 2005, 2008, 2012). The characteristic symptoms of PTSD resulting from exposure to war atrocities (criterion A) included re-experiencing the traumatic event (criterion B), avoiding stimuli associated with the trauma and experiencing a lack of general responsiveness (criterion C), and experiencing symptoms of increased arousal (criterion D). The full symptom picture must be present for more than one month and the disturbance must cause clinically significant distress or impairment in social, occupational, or other important areas of functioning (American Psychiatric Association [APA], 1994). The onset of the PTSD symptoms was assessed as part of the interview.

#### Emotion Dysregulation

Emotion dysregulation was assessed by the Difficulties in Emotion Regulation Scale Short Form (DERS-SF; Kaufman et al., 2015). The DERS-SF consists of six dimensions of emotion regulation wherein difficulties may occur, including (a) lack of awareness of emotional responses, (b) lack of clarity of emotional responses, (c) non-acceptance of emotional responses, (d) limited access to emotion regulation strategies perceived as effective, (e) difficulties controlling impulses when experiencing negative emotions, and (f) difficulties engaging in goal-directed behaviors when experiencing negative emotions. The DERS-SF consists of 18 items that maintains the excellent psychometric properties and retains the total and subscale scores of the original measure with half the items (Kaufman et al., 2015). Composite scores could range from 18 to 90, with higher scores indicating greater difficulties in emotion regulation (i.e., greater emotion dysregulation). Cronbach’s alpha for the total scale in this sample is 0.84.

#### Behavioral and Emotional Disorders

The Strengths and Difficulties Questionnaire (SDQ; Child Form) assessed behavioral and emotional disorders. The SDQ is a brief behavioral screening questionnaire that asks about 25 attributes, some positive and others negative (Goodman, 1997). The SDQ is administered to children to investigate their emotional and behavioral disorders. The 25 items are divided between five subscales of five items each, generating scores that assess conduct problems, inattention- hyperactivity, emotional symptoms, peer problems, and prosocial behavior; all scales but the last are summed to generate a Total Difficulties scores. Cronbach’s alpha for the total scale in this sample is 0.87.

### Statistical Analysis

Descriptive statistics were used to describe the basic features of the sample, followed by inter-correlations among war atrocities, neuroticism, and child’s developmental outcome measures. Then the potential mediator and moderator effect of neuroticism in the relationship between war atrocities and posttraumatic stress, emotion regulation and behavioral and emotional disorders were examined.

According to Baron and Kenny (1986), four statistical criteria are required to demonstrate a mediator effect. First, the predictor variable must be related to the outcome. Second, the mediator variable must be related to the outcome. Third, the predictor must be related to the mediator. Fourth, after controlling for the effects of the mediator on the outcome, the relation between the predictor and the outcome must be significantly reduced.

As for the moderator effects, three separate hierarchal regression analyses were performed to examine the interactive effects of war atrocities and neuroticism, in predicting the occurrence of PTSD, emotion dysregulation, and emotional and behavioral disorders in children. As recommended by Aiken and West (1991), the continuous variable war atrocities were centered (i.e., sample means were subtracted from each child’s score) before creating interaction terms. This technique is intended both to reduce multicollinearity and to facilitate interpretation of interaction terms.

## Results

### War Atrocities, Neuroticism, and Outcome Measures

Inter-correlations among war atrocities, neuroticism, and outcome measures are shown in Table 1. Pearson correlation results indicated that all study variables were positively correlated with each other, namely war atrocities, neuroticism, PTSD, emotion dysregulation, and emotional and behavioral disorders.

**TABLE 1:** Intercorrelations of predictor, mediator, and outcome variables.

		1	2	3	4	5
1.	War atrocities	–				
2.	Neuroticism	0.416**	–			
3.	PTSD	0.246**	0.314**	–		
4.	Emotion dysregulation	0.361**	0.648**	0.243**	–	
5.	Emotional and behavioral disorders	0.508**	0.557**	0.147**	0.537**	–

**Correlation is significant at the 0.01 level (2-tailed).

### Mediator Effects

#### War Atrocities, Neuroticism, and PTSD

The four-step model outlined by Baron and Kenny (1986) was followed to examine whether neuroticism was a mediating variable that accounts for the relationship between war atrocities and PTSD. As indicated in Table 2, all three relationships had significant positive correlations and met criteria for use in a mediational model. The standardized regression coefficient for the relationship between war atrocities and PTSD was significant (Criterion 1), and neuroticism was significantly related to PTSD (Criterion 2). In addition, the predictor variable war atrocities was related to neuroticism (Criterion 3). After controlling for the effects of neuroticism on PTSD, the relation between war atrocities and PTSD remained significant but partially mediated since the path from war atrocities to PTSD is reduced in absolute size. Thus, the results indicated that there is partial mediation: neuroticism was found to mediate the relationship between war atrocities and PTSD (Criterion 4).

**TABLE 2:** Predictor and the outcome variables before and after controlling for the effects of the mediator variable neuroticism.

Criteria	*B*	*SE B*	*t*	*p*
War atrocities → PTSD	0.246	0.004	8.032	0.000
Neuroticism → PTSD	0.314	0.003	10.430	0.000
War atrocities → neuroticism	0.416	0.034	14.462	0.000
War atrocities → PTSD after controlling for neuroticism	0.140	0.004	4.278	0.000
War atrocities → emotion dysregulation	0.361	0.084	12.222	0.000
Neuroticism → emotion dysregulation	0.648	0.058	26.869	0.000
War atrocities → neuroticism				
War atrocities → emotion dysregulation after controlling for neuroticism	0.110	0.075	4.192	0.000
War atrocities →emotional and behavioral disorders	0.508	0.046	18.632	0.000
Neuroticism → emotional and behavioral disorders	0.557	0.038	21.176	0.000
War atrocities → neuroticism				
War atrocities → emotional and behavioral disorders after controlling for neuroticism	0.334	0.046	12.408	0.000

#### War Atrocities, Neuroticism, and Emotion Dysregulation

The first model tested the direct effect of war atrocities on emotion dysregulation yielding a significant result. In addition, neuroticism was significantly related to emotion dysregulation. Next, both war atrocities and neuroticism were entered into a regression equation. The standardized coefficients (β) for war atrocities and neuroticism were significant. After controlling for the effects of neuroticism symptoms on emotion dysregulation, the relation between war atrocities and emotion dysregulation remained significant but was reduced in absolute size. Thus, the relationship between war atrocities and emotion dysregulation was partially mediated by neuroticism (Table 2).

#### War Atrocities, Neuroticism, and Emotional and Behavioral Disorders

As depicted in Table 2, zero-order correlations between the war atrocities and the emotional and behavioral disorder measure (SDQ) indicated that war atrocities were significantly related to emotional and behavioral disorders (criterion 1), and neuroticism symptoms were significantly related to emotional and behavioral disorders (criterion 2). In addition, war atrocities were related to the neuroticism. Fourth, after controlling for the effects of the mediator on the outcome, the relation between the predictor and the outcome must be significantly reduced.

### Moderator Effects

#### War Atrocities, Neuroticism, and PTSD

Results of the hierarchal regression models are presented in Table 3. In the first step, war atrocities accounted for 6.1% of the variance in PTSD symptoms among refugee children. Adding neuroticism in the second step did produce a significant increase (5.4%) in the amount of variance in PTSD, neuroticism did directly predict PTSD. In the third regression step, the addition of war atrocities × neuroticism interaction term to the additive model of war atrocities and neuroticism did yield a significant change in *R*^2^ (2.1%). The moderator effects are indicated by the significant effect of war atrocities × neuroticism while war atrocities and neuroticism are controlled. Thus, neuroticism did moderate the effect of war atrocities on PTSD symptoms in refugee children (see Table 3).

**TABLE 3:** Hierarchical regression models predicting the occurrence of PTSD, emotion dysregulation, and emotional, and behavioral disorders in refugee children.

Criteria	*beta*	*R* ^2^	*t*	*p*
***PTSD***				
Step 1				.
War atrocities	0.25	0.060	8.03	0001
Step 2				
War atrocities	0.14	0.113	4.27	0.0001
Neuroticism	0.26		7.78	0.0001
Step 3				
War atrocities	0.10	0.133	3.06	0.002
Neuroticism	0.30		8.91	0.0001
War atrocities × neuroticism	0.15		4.97	0.0001
***Emotion dysregulation***				
Step 1				
War atrocities	0.36	0.130	12.22	0.0001
Step 2				
War atrocities	0.11	0.430	4.19	0.0001
Neuroticism	0.60		22.88	0.0001
Step 3				
War atrocities	0.11	0.430	4.09	0.0001
Neuroticism	0.60		8.01	0.0001
War atrocities x neuroticism	–0.00		–0.05	0.95
***Emotional and behavioral disorders***				
Step 1				
War atrocities	0.51	0.258	18.63	0.0001
Step 2				
War atrocities	0.33	0.402	12.40	0.0001
Neuroticism	0.42		15.51	0.0001
Step 3				
War atrocities	0.35	0.404	12.50	0.0001
Neuroticism	0.40		14.47	0.0001
War atrocities × neuroticism	–0.04		–1.81	0.07

#### War Atrocities, Neuroticism, and Emotion Dysregulation

Table 3 display results of the moderating role of neuroticism in the relation between war atrocities and emotion dysregulation. In the first step, war atrocities accounted for 13% of the variance in emotion dysregulation among refugee children. Adding the neuroticism variable in the second step did produce a significant increase (30%) in the amount of variance in emotion dysregulation. In the third regression step, the addition of the war atrocities × neuroticism interaction term to the additive model did not yield significant results.

#### War Atrocities, Neuroticism, and Emotional and Behavioral Disorders

A similar pattern was found for child reports of emotional and behavioral disorders. In the first step, war atrocities accounted for 25.8% of the variance in emotional and behavioral disorders among refugee children. Adding the neuroticism variable in the second step did produce a significant increase (14.4%) in the amount of variance in emotional and behavioral disorders. Addition of the war atrocities × neuroticism interaction term to the additive model of war atrocities and neuroticism resulted in non-significant change in *R*^2^ (0.002) indicating that the moderating role of neuroticism was not supported (see Table 3).

## Discussion

Several important findings emerged from this study of the relations among war atrocities, neuroticism, and psychopathological symptoms in Syrian refugee children who resettled in Lebanon and Jordan. First, war atrocities significantly predicted children’s PTSD, emotion dysregulation, and emotional and behavioral disorders. These associations are similar to earlier reports showing that children who experience higher levels of war atrocities report higher levels of psychopathological problems (e.g., Khamis, 2005, 2008, 2012; Masten and Narayan, 2012). Second, results of the current study revealed a partial mediating effect of neuroticism on PTSD, emotion dysregulation, and emotional and behavioral disorders. In addition, the findings revealed full moderating effects of neuroticism on PTSD as well as partial moderating effects on emotion dysregulation, and emotional and behavioral. These findings emphasize that increased levels of neuroticism symptoms following exposure to war atrocities, may result in the promulgation of symptoms beyond what could be explained by direct effects alone. This is supported not only by the partial mediation effect found in each of the models, but also reinforced by other studies showing high rates of comorbidity between neuroticism and all forms of psychopathology (Santor and Rosenbluth, 2005; Kotov et al., 2010) including PTSD *(Hasanović, 2012; Breslau and Schultz, 2013)*, emotion dysregulation (Kokkonen and Pulkkinen, 2001; Weinstock and Whisman, 2006; Mącik et al., 2019), and emotional and behavioral disorders (e.g., Khan et al., 2005; Martel and Nigg, 2006; Lahey, 2009).

Overall, the data from this study reveal that neuroticism plays a pivotal role in the development of psychopathological symptoms in refugee children. The strong positive relationship between neuroticism and the various developmental outcomes in this sample provide some challenges for theoretical models. A primary implication of this study highlights the importance of exploring whether neuroticism increases as a function of PTSD symptoms, emotion dysregulation, and emotional and behavioral disorders; whether neuroticism and psychopathological symptoms reciprocally predict each other, or whether the relation is spurious because of third variables that affect both neuroticism and psychopathological symptoms. Additional prospective studies are needed to understand the mechanisms and the processes through which elevated levels of neuroticism lead to greater PTSD symptom severity, emotion dysregulation (ED) and emotional and behavioral disorders (EBD) in children. Recognition of such variables could improve prevention and intervention programs, which typically focus on targeted mindfulness interventions (Drake et al., 2017), emotion regulation training (Ogedegbe et al., 2012).

## Strengths, Limitations, and Future Directions

This study has numerous strengths: a quantitative research design, a large sample drawn from two host countries with different geographical backgrounds, four measures of mental health outcomes, and a focus on refugee school-age children. In addition, the sample characteristics demonstrate diversity in terms of gender, age, and level of education. However, conclusions based on this study should be guided by limitations of the research design. First, the study design is cross-sectional, and the data are correlational. Thus, no definitive statements regarding causality can be made. Second, as this is a sample of school-age children, the findings cannot be generalized to the overall population of refugee children (e.g., out-of-school children). Further empirical inquiry is required to determine whether the findings of this study will generalize to unregistered refugees and to unaccompanied refugee children who resettled in other countries and cultures with distinct backgrounds and traditions. Third, analyses of the relationship between trauma and psychopathological outcomes were limited to war atrocities, and thus, the pattern of results found may not generalize to refugee children who may have been exposed to other, possibly more difficult, flight trajectories, marked by sexual abuse, forced marriage and prostitution (Ward and Marsh, 2006), as well as continued exposure to sexual maltreatment within host countries (Lay and Papadopoulos, 2009). Longitudinal research is needed for elucidating the directionality of the relationship between post-resettlement traumas and developmental outcomes.

## Conclusion

The present study contributes to the neuroticism literature by showing that high-N children develop more problems that are psychopathological and have more severe affective reactions to war atrocities in post- resettlement contexts. These findings help to explain the chronic negative affectivity experienced by high-N children. Thus, incorporating personality assessment into treatment planning might lead to more personalized treatment plans that will improve clinical outcomes (Widiger and Presnall, 2013). Early interventions aimed to reduce neuroticism might contribute to a better prognosis in refugee children who are at high-risk for psychological disorders. Neuroticism may also represent a potential future target for intervention for children/adolescents to promote their mental health by strengthening resiliency.

## Data Availability Statement

The raw data supporting the conclusions of this article will be made available by the authors, without undue reservation.

## Ethics Statement

The studies involving human participants were reviewed and approved by American University of Beirut-IRB. Written informed consent to participate in this study was provided by the participants’ legal guardian/next of kin.

## Author Contributions

The author confirms being the sole contributor of this work and has approved it for publication.

## Conflict of Interest

The author declares that the research was conducted in the absence of any commercial or financial relationships that could be construed as a potential conflict of interest.

## Publisher’s Note

All claims expressed in this article are solely those of the authors and do not necessarily represent those of their affiliated organizations, or those of the publisher, the editors and the reviewers. Any product that may be evaluated in this article, or claim that may be made by its manufacturer, is not guaranteed or endorsed by the publisher.
